# Editorial: Neuro-glia-immune communication: novel biomarkers and therapeutic targets

**DOI:** 10.3389/fncel.2023.1287049

**Published:** 2023-09-29

**Authors:** Fernanda Gubert, Camila Zaverucha-do-Valle, Louise A. Mesentier-Louro, Pedro Moreno Pimentel-Coelho

**Affiliations:** ^1^Instituto de Ciências Biomédicas - Universidade Federal do Rio de Janeiro, Rio de Janeiro, Brazil; ^2^Instituto Nacional de Infectologia Evandro Chagas, Fundação Oswaldo Cruz, Rio de Janeiro, Brazil; ^3^Nash Family Department of Neuroscience, Ronald M. Loeb Center for Alzheimer's Disease, and Friedman Brain Institute, Icahn School of Medicine at Mount Sinai, New York, NY, United States; ^4^Instituto de Biofísica Carlos Chagas Filho, Universidade Federal do Rio de Janeiro, Rio de Janeiro, Brazil

**Keywords:** neuro-glia-immune communication, neurodegenerative diseases, microglia, stroke, GABAergic neurons

Neuronal injury and loss remain untreatable consequences of damage to the central nervous system (CNS), which can occur in common neurological and neurodegenerative conditions like multiple sclerosis (MS), Alzheimer's disease (AD), stroke, Parkinson's disease (PD), and others. Neurodegenerative diseases are the result of disruptions in different cells, not only in neurons, and even in different systems beyond the nervous system. The understanding of how non-neuronal cells contribute to neurodegenerative phenotypes will unravel novel mechanisms of disease and support the discovery of more efficient treatments. The goal of this Research Topic was to foster a discussion about the role of non-neuronal cells and systemic clues as key mediators of neuronal fate, and to provide new tools to study non-neuronal cell phenotypes in neurodegenerative conditions.

MS is the most prevalent neurodegenerative disease in young adults and is associated with complex interactions between the immune and central nervous systems, involving several cell types. The predominant view is that activation and infiltration of peripheral immune system cells into the CNS lead to demyelination and neurodegeneration (Ransohoff et al., 2015). Although MS is a classic example of a neurodegenerative disease caused by peripheral immune cells, resident neuroinflammatory cells like astrocytes and microglia are also involved in the progression of the disease. Using the experimental autoimmune encephalomyelitis (EAE) mouse model of MS, Hoelz et al. combined two pharmacological approaches, dimethyl fumarate and pregabalin, to target peripheral immune cells, microglia, and astrocytes. This combined therapy significantly attenuated MS clinical symptoms, which was not observed using single approaches. The positive effect was correlated with a reduced glial response in EAE, suggesting that astrocytes and microglia contribute to MS pathophysiology and supporting the investigation of glia-targeting therapy in MS. This study contributes to the view that combined approaches targeting immune cells and neuroinflammation will promote more efficient neuroprotection in MS, including at chronic stages of the disease.

In contrast with the impact of MS in the young population, the world population is growing older and neurodegenerative disorders are a major concern. AD is the most common type of dementia and is characterized by the accumulation of amyloid plaques and tau neurofibrillary tangles in the brain. It is widely accepted that AD is associated with a combination of genetic, environmental and lifestyle risk factors (Armstrong, 2019). As a result, many studies have been focusing on developing preventative strategies and treatment approaches based on lifestyle changes. There is growing evidence that physical exercise alleviates memory impairment in AD, which has led to further investigation into the cellular and molecular mechanisms of exercise. Lourenço et al. showed that an exercise-induced myokine, irisin, prevented amyloid-β oligomers-induced oxidative stress in hippocampal neurons *in vitro*, which was associated with increased brain-derived neurotrophic factor (BDNF) levels and extracellular signal-regulated kinase (ERK) activation. Interestingly, in postmortem hippocampal samples from subjects over 90 years old and from subjects with high tau pathology, there was a trend of reduced expression of fibronectin type III domain containing protein 5 (FNDC5), which is the irisin precursor. While the effect of irisin in other cell types, like glial cells, is still elusive, the findings of this study suggest that irisin has a neuroprotective effect in hippocampal neurons and that irisin-mediated signaling pathways can reveal novel therapeutic targets for AD.

The study of neurodegenerative diseases and neuroinflammation often involves analyzing and comparing the level of microglial activation in different conditions. During activation, microglia undergo morphological changes going from a homeostatic ramified cell to an ameboid activated cell with multiple stages in between (Paolicelli et al., 2022). It is still challenging to quantify the level of activation based on morphological changes using an unbiased method. For that reason, Stetzik et al. developed an automated accurate method to identify microglia and their changes in morphology and reactivity using commercially available deep learning tools (Aiforia^®^). By using this method, the authors quantified changes in microglia morphology in mouse models of viral infection and α-synuclein aggregation and the obtained results were consistent with previous reports. The described method was validated at or above human performance and could help to optimize data on microglia activation.

Although glial response is commonly associated with a worse outcome, studies investigating glial-targeted treatments should consider that glial cells have an important role in maintaining CNS homeostasis in health and disease. Attenuating glial responses can have differential and even detrimental effects depending on the disease stage, comorbidities, as well as genetic and environmental risk factors. These phenomena can be exemplified with microglia. Microglia can adopt different phenotypes or states in pathological conditions, including neurodegenerative diseases (Paolicelli et al., 2022). Modulation of microglial function by enhancing their homeostatic and repairing functions, while decreasing their neurotoxic potential, offer a promising approach to treat neurological disorders such as AD. In a mini-review article, Revuelta et al. present current evidence demonstrating that microglial function is also controlled by ion channels. They discuss how inhibitors of the voltage-gated K^+^ channel K_V_1.3 can produce protective effects in models of AD by modulating microglial activation.

Besides neurodegenerative disorders, glial cells play an important role in acute lesions to the CNS, such as stroke. In 2019 it was estimated that 12.2 million people suffered a stroke worldwide and 6.6 million died from stroke, making it the second leading cause of death in the world (GBD 2019 Stroke Collaborators, 2019). After the primary insult and the following neuronal death, the inflammatory cascade initiates to isolate the injured area and limit the damage. However, the magnitude of this immune response usually contributes to the secondary damage of the injured area. In this scenario, understanding how the different components of the inflammatory response contribute to preventing neuronal death or increase damage is essential to the development of clinical strategies. The neurotransmitter gamma-aminobutyric acid (GABA) is the main inhibitory neurotransmitter in the brain and besides its well-established role on neuronal cells, many groups are studying its role on non-neuronal cells, especially after injury. Functional GABAA receptors have been described in astrocytes, microglia, dendritic cells, T cells, natural killer cells, monocytes, B cells and neutrophils—the main players in the inflammatory cascade that follows stroke. In their review, Michalettos and Ruscher approach the immunomodulatory actions of GABA and how they affect astrocytes, microglia and peripheral immune cells after stroke.

The original articles and reviews present in this Research Topic demonstrate the complexity of neurodegenerative and acute injuries in the CNS, and highlight the importance of exploring beyond the classical pathological pathways to achieve more efficient therapeutic results. Glial cells, like microglia, are relevant targets for treatments. However, it is important to understand the duality of their functions and to improve methods for analyzing them. Investigating other neuronal types, such as inhibitory GABAergic neurons, can also reveal how the multicellular environment contributes to disease phenotypes. Ultimately, it is important to remember that all systems are integrated and that lifestyle factors, like exercise, can have a major influence on neurodegenerative diseases. We thank all authors and reviewers who contributed to this Research Topic and increased our knowledge about the complex neuro-glia-immune communication.

## Author contributions

FG: Writing—original draft. CZ-d-V: Writing—original draft. LM-L: Writing—review and editing. PP-C: Writing—original draft.
